# Physical activity-equivalent label reduces consumption of discretionary snack foods

**DOI:** 10.1017/S1368980018000228

**Published:** 2018-03-01

**Authors:** Isabella E Hartley, Russell SJ Keast, Dijn G Liem

**Affiliations:** Deakin University, Centre for Advanced Sensory Science, School of Exercise and Nutrition Sciences, Burwood, VIC 3125, Australia

**Keywords:** Physical activity, Food labelling, Energy intake, Snack consumption, Discretionary food

## Abstract

**Objective:**

The present research aimed to investigate the impact of the physical activity calorie equivalent (PACE) front-of-pack label on consumption, prospective consumption and liking of familiar and unfamiliar discretionary snack foods.

**Design:**

In a within-subject randomised design, participants tasted and rated liking (9-point hedonic scale) and prospective consumption (9-point category scale) of four different snack foods with four different labels (i.e. blank, fake, PACE, PACE doubled) and four control snack foods. The twenty snack foods were presented during two 45 min sessions (i.e. ten snack foods per session) which were separated by one week. The amount participants sampled of each snack food was measured.

**Setting:**

The study was conducted in the Centre for Advanced Sensory Sciences laboratory at Deakin University, Australia.

**Subjects:**

The participants were 153 university students (126 females, twenty-seven males, mean age 24·3 (sd 4·9) years) currently enrolled in an undergraduate nutrition degree at Deakin University.

**Results:**

When the PACE label was present on familiar snack foods, participants sampled 9·9 % (22·8 (sem 1·4) *v.* 25·3 (sem 1·5) g, *P*=0·03) less than when such label was not present. This was in line with a decreased prospective snack food consumption of 9·1 % (3·0 (sem 0·2) *v.* 3·3 (sem 0·2) servings, *P*=0·03). Such pattern was not seen in unfamiliar snacks.

**Conclusions:**

The PACE label appears to be a promising way to decrease familiar discretionary snack food consumption in young, health-minded participants.

Overweight and obesity threatens the health of six in ten adults in Australia. This is associated with an estimated cost of $AU 56·6 billion^(^ ^1^ ^)^ in health-care bills, government subsidies and lost productivity due to the adverse health outcomes related to overweight and obesity, such as CVD, hypertension and diabetes^(^ ^2^ ^)^. The average weight of males and females has increased substantially since 1995 (by 3·9 and 4·1 kg, respectively)^(^ ^3^ ^)^. Currently 35·5 % of Australians are considered overweight and 27·9 % considered obese^(^ ^4^ ^)^, and it is predicted that the number of obese Australians will increase to 35 % by 2025^(^ ^5^ ^)^. Overweight and obesity is caused by a positive energy imbalance that is often small, however accumulates over time to result in weight gain^(^ ^6^ ^)^. It has been calculated that decreasing energy intake and/or increasing physical activity by 418 kJ/d may be sufficient to prevent weight gain in the population^(^ ^7^ ^)^.

The positive effect of decreasing energy intake by reducing consumption of discretionary foods would see more recommendations for a healthy diet being met. Discretionary foods are defined as foods typically containing high levels of saturated fat, refined sugar and/or salt, while providing little nutritional value^(^ ^8^ ^)^. In Australia, people consume more than half a kilogram of discretionary foods per day, making up on average 35 % of daily energy intake^(^ ^8^ ^)^. Among these discretionary foods are unhealthy snack foods such as chips/crisps, other salty snacks, cakes, sweet biscuits, ice cream and chocolate^(^ ^9^ ^,^ ^10^ ^)^. Consumption surveys suggest that on average 10·5 % of Australian adults’ daily energy intake (approximately 914 kJ) comes specifically from these discretionary snack foods^(^ ^11^ ^)^.

There are various factors that drive individuals’ consumption of snack foods. A study assessing individuals’ motives to consume unhealthy snacks found that in 55 % of snacking occasions, participants cited the snack ‘looked or smelt tempting’, hunger (49 %), to avoid being hungry later (22 %) and the need for energy (23 %)^(^ ^12^ ^)^. Furthermore, consumption of unhealthy snacks that contain fat, sugar and high levels of sodium is often seen in the absence of hunger^(^ ^13^ ^)^. It has been suggested that subtly guiding consumers’ food choice by providing cues such as front-of-pack (FOP) labelling can prompt healthier food choices^(^ ^14^ ^)^. Labelling can influence food choices through providing information regarding the energy and nutrient content, thereby assisting consumers to make less energy-dense food choices^(^ ^15^ ^,^ ^16^ ^)^. Whether kilojoule information alone is enough to affect food choice is controversial. Some studies have found a reduction in kilojoules ordered in online restaurant settings^(^ ^17^ ^)^, in less health-conscious participants^(^ ^18^ ^)^, only in lean females^(^ ^19^ ^)^ or not at all^(^ ^20^ ^)^ when kilojoule information was presented. Systematic reviews have found that kilojoule information alone may not be the most effective labelling format to lower unhealthy food choice and consumption^(^ ^15^ ^,^ ^16^ ^)^. This may be in part due to many consumers struggling to understand what kilojoules are in the context of their overall health and that a certain level of knowledge is required to interpret these labels^(^ ^21^ ^,^ ^22^ ^)^. A simple FOP label that can be easily understood by all demographics in the population may be more effective at reducing discretionary snack food consumption.

Recently, the Royal Society for Public Health in the UK called for an activity-equivalent label to be implemented as a FOP labelling format^(^ ^23^ ^)^. The physical activity calorie equivalent (PACE) label is an exercise-equivalent FOP label that displays, as a pictogram, the number of minutes of walking (or other forms of exercise) required to burn off the kilojoules in one serving of a food. There are different designs of PACE labels, some may also include kilojoule information per serving alongside the exercise-equivalent information^(^ ^24^ ^–^ ^26^ ^)^. Other activity-equivalent labels have previously been referred to as a physical activity-based label^(^ ^27^ ^)^ or a physical activity label^(^ ^28^ ^)^. In the present study, we consider a PACE label as any label that displays a pictogram representing the amount of physical activity required to burn off the kilojoules in a serving of food. In controlled studies where participants ordered fast foods^(^ ^24^ ^,^ ^27^ ^)^ and snack foods^(^ ^28^ ^)^ online, it was found that the presence of a PACE-type label (presented as either minutes or miles to walk) significantly reduced the number of kilojoules participants ordered. Similarly, intervention studies found that presenting the PACE information at the point of sale significantly reduced selection^(^ ^29^ ^)^ and purchasing^(^ ^30^ ^)^ of sugar-sweetened beverages. Only two previous studies have assessed the impact of a PACE-style label on food choice and consumption^(^ ^26^ ^,^ ^31^ ^)^. In a controlled setting, it was found that the numbers of kilojoules ordered and consumed from a fast-food lunch menu were reduced in the presence of written exercise-equivalent information^(^ ^31^ ^)^. Similarly, it was found that providing participants with PACE information (including kilojoule information) prior to watching a 30 min video significantly reduced the amount of cheese crackers participants consumed over the duration of the video screening^(^ ^26^ ^)^. Furthermore, there is evidence that the PACE label is preferred by consumers over kilojoule labelling, with one study finding 82 % of participants preferred the PACE label^(^ ^27^ ^)^. Qualitative studies have also found that participants were able to interpret the PACE label easily and believed that the PACE label would assist them to apply the foods’ kilojoule information into their food choices^(^ ^32^ ^)^. These studies suggest that the PACE label may be effective at lowering kilojoule ordering and purchasing^(^ ^24^ ^)^, consumption of fast food^(^ ^31^ ^)^ and is preferred and understood by consumers^(^ ^32^ ^)^. Additionally, it has been suggested that consumers are less likely to look at labels of products they are familiar with or consume regularly^(^ ^22^ ^)^, but may pay attention to FOP labels presented on novel/unfamiliar brands^(^ ^33^ ^)^. The previous studies have investigated readily available, regularly consumed fast foods, sugar-sweetened beverages and snack foods^(^ ^29^ ^,^ ^24^ ^,^ ^27^ ^,^ ^28^ ^,^ ^26^ ^,^ ^31^ ^)^. Whether the same effect would be seen in both familiar and unfamiliar snack foods has not previously been investigated. The influence of the PACE label on actual consumption and prospective consumption of both familiar and unfamiliar discretionary snack foods remains unclear.

In the present study, we examined whether the presence of the PACE label influenced consumption (how much participants sampled), prospective consumption and liking of discretionary snack foods, for both familiar and unfamiliar snacks. We hypothesised that the PACE label would lead to lower consumption than the non-PACE labels.

## Materials and methods

### Overall study design

Participants came to the Centre for Advanced Sensory Science laboratory at Deakin University, Burwood, Australia, for two sessions separated by one week. During each session they tasted and rated liking and prospective consumption of ten snack foods which differed in sensory profile and labelling. The amount which was tasted was measured for all snack foods.

### Participants

Recruited participants (*n* 153) were students enrolled in an undergraduate nutrition degree at Deakin University. Twenty students (seventeen females and three males, mean age 22·8 (sd 2·3) years) failed to complete both sensory tasting sessions and were excluded from the final analyses. Students with food allergies or intolerances were excluded from participation.

### Snack products

Twenty-four snack products were initially evaluated in a qualitative pilot tasting with five members of the Centre for Advanced Sensory Science. These products were evaluated on familiarity and liking as well as their overall flavour and texture profile. From this pilot two commercially available familiar snacks foods (Nacho Cheese Shapes (Arnotts, Australia) and Honey Cashews (Coles Scoop n’ Weigh), both sold at mainstream supermarkets) and two unfamiliar snack foods (Shrimp Peanut Crackers (Khao Shong, Thailand) and Cheese Rice Crackers (Want Want, Taiwan), both sold at speciality Asian stores) were selected to approximate flavour and texture, resulting in the selection of two crunchy nut snacks and two cheese biscuit snacks (see Table 1). A commercially available, moderately liked dummy snack was also included in the study to minimise first order effect (Seaweed Rice Cracker (Fantastic, Thailand)). Samples were presented in a 25 g serving size, on a white plate (BioPak, 180 mm diameter), with a corresponding photo (14 cm×21 cm) of the snack product presented on touch-screen monitors (Dell, model number 52240Tb).

**Table 1:** (colour online) Description of snacks used in the present study: number of minutes required to burn off the kilojoules in the 25 g serving size, number of kilojoules per 25 g serving and amount of total fat, saturated fat and sodium per 25 g serving size

Snack photo	Snack name	Minutes walking to burn 25 g serving	Kilojoules per 25 g serving	Total fat (g) per 25 g serving	Saturated fat (g) per 25 g serving	Na (mg) per 25 g serving
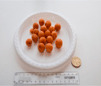	Shrimp Peanut Crackers	30	490	3·6	1·6	111
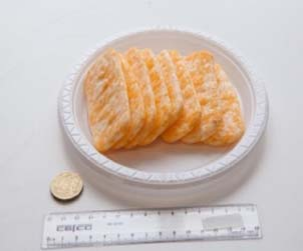	Cheese Rice Crackers	32	512·5	5·8	2·9	250
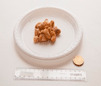	Honey Cashews	35	567	8·5	1·6	3·3
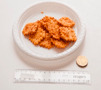	Nacho Cheese Shapes	32	525	4·3	1·2	205·5
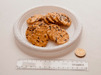	Seaweed Rice Crackers	23	380	0·1	0·0	129

### Labels

**Fig. 1:**
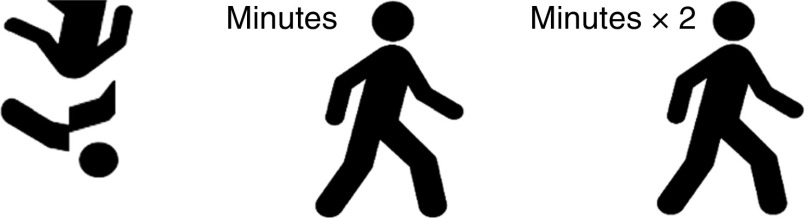
Left to right: pictograms of Fake, PACE and PACE×2 labels presented on snack photos (PACE, physical activity calorie equivalent; PACE×2, PACE label with walking minutes doubled)

The label design was derived from previous studies that have used a PACE-style label in minutes^(^ ^24^ ^,^ ^27^ ^)^. Four labels were presented once on each of the four snacks and dummy snack, to total twenty snack/label variations. As shown in Fig. 1, labels included were: Blank (no label); Fake (equivalent proportion of black and white as the PACE labels); PACE walking minutes; and PACE walking minutes doubled (PACE×2). Labels (5 cm×4·5 cm) were presented in the upper right-hand corner of the snack photograph presented to participants on the touch-screen monitors (see Table 2). The second PACE label representing double the number of minutes was included to estimate a dose–response effect of PACE on the outcome variables. The minutes presented on the PACE label were calculated based on the metabolic equivalent intensity level of a 74 kg adult walking (3 MET)^(^ ^34^ ^)^ and the kilojoule content of one 25 g serving of the snack food.

**Table 2:** (colour online) Labels that were presented to participants during tasting; each snack/label combination was presented to participants as they were given the corresponding sample to taste

	Label			
	Blank	Fake	PACE	PACE×2
Snacks 5×25 g servings, individually served				
Honey Cashews	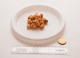	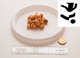	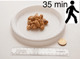	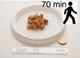
Nacho Cheese Shapes	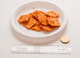	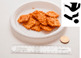	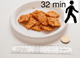	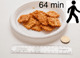
Cheese Rice Crackers	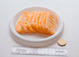	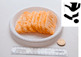	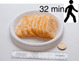	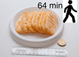
Shrimp Peanut Crackers	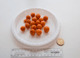	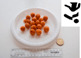	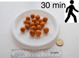	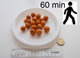
Seaweed Rice Crackers	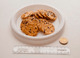	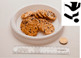	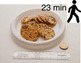	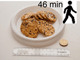

PACE, physical activity calorie equivalent; PACE×2, PACE label with walking minutes doubled.

### Experimental design

Participants came to the Centre for Advanced Sensory Science once per week at the same time and day for two consecutive weeks. Tasting took place in individual partitioned sensory booths, which minimised interaction between the participants during tasting of the snack foods. At the commencement of each session participants were given the following instructions: ‘You will be presented with a number of snack foods. These snack foods will also be shown on a photo on your screen. In each photo you might also see a pictogram of a man walking. This represents the number of minutes you would need to walk to burn the amount of snack food that you are presented. You can eat as much of the snack foods as you want. Please do not overthink your answers and just provide the answer that first comes to mind’. While tasting the snacks, participants were asked to rate the snack food on liking, prospective consumption and familiarity. Between individual snacks participants had a 2 min break where they were instructed to have a sip of water and a bite of an unsalted cracker (Coles, Australia). Each combination of label and snack was given once (five snacks×four labels) over two consecutive weeks. Each week consisted of two sampling sessions within 45 min and sampling sessions were separated by a 5 min break, to total four sampling sessions over two weeks. Within each sampling session participants were presented with all test snacks (Shrimp Peanut Crackers, Cheese Rice Crackers, Honey Cashews, Nacho Cheese Shapes) once in a randomised order, and the dummy sample (Seaweed Rice Crackers), which was presented as the first sample for each of the four sampling sessions to minimise first order effect. Each snack was assigned a randomised label (e.g. Blank, Fake, PACE, PACE×2) and each snack/label combination was assigned a randomised unique three-digit code, which was visible on the plate the snack was presented to participants on. In total, participants tasted twenty snack/label variations in a randomised order over two sessions in two consecutive weeks.

### Outcome measures

Outcome measures were liking, prospective consumption, actual consumption (how much participants sampled) and familiarity. Liking was measured on a 9-point hedonic scale ranging from 1=‘dislike extremely’ to 9=‘like extremely’. Prospective consumption was measured by participants selecting the number of servings (i.e. 0, 0·5, 1, 1·5, 2, 2·5, 3, 3·5, more than 3·5) they thought they would eat of the snack ‘right now’. Familiarity was measured in an online survey containing the same photos of the snacks as the tasting sessions, which participants completed prior to attending the tasting sessions. Familiarity was rated on a 5-point Likert scale ranging from 1=‘not familiar at all’ to 5=‘very familiar’. Liking, prospective consumption and familiarity data were collected using Compusense Cloud Software as part of the Compusense Academic Consortium. How much participants sampled was measured to 0·2 g (Ohaus, model number NV 4101) by subtracting the post-tasting weight from the pre-tasting weight; this was recorded manually on participants’ individual slips.

### Statistical analysis

Participants who did not complete both tasting sessions were excluded from analysis. Consumption data were excluded if ≤0·000 g, as negative consumption was not possible. Liking data associated with consumption data ≤0·000 g were also excluded as participants were not able to rate taste liking without sampling any of the snack presented to them. Familiarity data were collected via an online survey that participants completed prior to attending tasting sessions.

To assess differences in overall consumption, prospective consumption, familiarity and liking of the test snacks and label combinations, a Friedman analysis for ranks was conducted with statistical significance set at *P*<0·05. Where the Friedman analysis was significant, a Wilcoxon signed-ranks test was then used to find which label was significantly different within the test snacks. Within each snack food, six different comparisons were made (i.e. Blank *v.* Fake, Blank *v.* PACE, Blank *v.* PACE×2, Fake *v.* PACE, Fake *v.* PACE×2, PACE *v.* PACE×2); therefore the significance levels for these comparisons was set at 0·05/6=0·008 (*P*<0·008).

To assess the impact of the PACE labels (PACE and PACE×2) compared with non-PACE labels (Blank and Fake), snacks were combined into two groups based on familiarity and how much participants sampled (in grams). Honey Cashews and Nacho Cheese Shapes were grouped into the familiar category, and Shrimp Peanut Crackers and Cheese Rice Crackers were grouped into the unfamiliar category. This was an *a priori* classification where two familiar and unfamiliar snacks were selected. A Wilcoxon signed-ranks test was conducted to determine whether participants’ familiarity rating was in fact higher for the selected familiar snacks, with significance set at *P*<0·05. A Wilcoxon signed-ranks test was then conducted to compare PACE label *v*. non-PACE label for consumption, prospective consumption and liking, with significance set at *P*<0·05.

## Results

### Participant characteristics

A total of 153 students participated in the study, 126 (82·4 %) of whom were female (mean age 24·1 (sd 4·7) years) and twenty-seven (17·6 %) of whom were male (mean age 25·1 (sd 5·8) years). Mean BMI of females was 22·5 (sd 3·2) kg/m^2^ and of males was 25·2 (sd 4·7) kg/m^2^ (see Table 3). ‘Consumption’ in the remainder of the ‘Results’ section refers to the amount participants sampled while tasting snack foods.

**Table 3:** Demographic information of participants in the snack study: university students (n 153) enrolled in an undergraduate nutrition degree at Deakin University, Australia

	Female		Male	
	*n*	%	*n*	%
Sex	126	82·4	27	17·6
Age (years)				
Mean	24·1		25·1	
sd	4·7		5·8	
BMI (kg/m^2^)				
Mean	22·5		25·2	
sd	3·2		4·7	
Nationality				
Australian	76	60·3	10	37·0
Asian	23	18·3	14	51·9
Other	17	13·5	1	3·7
Missing nationality data	10	7·9	2	7·4

### Overall consumption, prospective consumption and familiarity

Snack foods that were assumed to be familiar were indeed perceived more familiar than the snack foods that were assumed not to be familiar (3·8 (sd 1·0) *v.* 2·4 (sd 1·3), *P*<0·01). Consumption and prospective consumption were lower for Shrimp Peanut Crackers compared with all the other snack foods tested (all *P*<0·001). Liking, prospective consumption and consumption (in grams) were significantly higher overall for familiar snacks compared with unfamiliar snacks (*P*=0·000; see Table 4).

**Table 4:** Mean consumption, liking, prospective consumption and familiarity results for each of the four test snack snacks (Shrimp Peanut Crackers, Honey Cashews, Cheese Rice Crackers and Nacho Cheese Shapes) and labels (Blank, Fake, PACE and PACE×2) among 153 university students (126 females, twenty-seven males, mean age 24·3 (sd 4·9) years) enrolled in an undergraduate nutrition degree at Deakin University, Australia

	Consumption (g)		Liking rating		Prospective consumption (no. of servings)		Familiarity rating	
Snack/label combination	Mean	sd	Mean	sd	Mean	sd	Mean	sd
Shrimp Peanut Crackers/Blank	2·9^a^	3·3	3·8^a^	2·3	0·3^a^	0·5	2·4	1·3
Shrimp Peanut Crackers/Fake	2·9^a^	3·2	4·1^a^	2·3	0·3^a^	0·4		
Shrimp Peanut Crackers/PACE	3·5^a^	4·0	4·1^a^	2·3	0·3^a^	0·5		
Shrimp Peanut Crackers/PACE×2	3·2^a^	3·5	4·0^a^	2·3	0·2^b^	0·4		
Honey Cashews/Blank	5·8^a^	4·9	6·4^a^	1·9	0·8^a^	0·7	3·8	1·0
Honey Cashews/Fake	6·1^a^	4·8	6·6^a^	1·8	0·8^a^	0·7		
Honey Cashews/PACE	5·4^a^	4·6	6·3^a^	2·0	0·7^a^	0·6		
Honey Cashews/PACE×2	5·6^a^	5·2	6·5^a^	1·8	0·7^a^	0·6		
Cheese Rice Crackers/Blank	4·6^a^	4·8	5·8^a^	2·3	0·5^a^	0·5	3·1	1·2
Cheese Rice Crackers/Fake	4·5^a^	4·9	5·7^a^	2·1	0·5^a^	0·5		
Cheese Rice Crackers/PACE	4·7^a^	4·9	5·8^a^	2·2	0·5^a^	0·5		
Cheese rice Crackers/PACE×2	4·7^a^	4·8	5·7^a^	2·1	0·5^a^	0·6		
Nacho Cheese Shapes/Blank	6·4^a^	5·4	6·7^a^	6·7	0·9^a^	0·7	4·0	0·9
Nacho Cheese Shapes/Fake	6·4^a^	5·6	6·7^a^	6·7	0·9^a^	0·8		
Nacho Cheese Shapes/PACE	6·3^a^	5·8	6·8^a^	1·6	0·9^a^	0·7		
Nacho Cheese Shapes/PACE×2	6·1^a^	5·0	6·8^a^	1·6	0·8^a^	0·7		

PACE, physical activity calorie equivalent; PACE×2, PACE label with walking minutes doubled. Friedman analysis was conducted within each snack type, with significance set at *P*<0·05. Where significant, the Wilcoxon signed-ranks test was was conducted, with significance set at *P*<0·008. ^a,b^Within each snack type, mean values within a column with unlike superscript letters were significantly different (*P*<0·008).

### PACE labels v. non-PACE labels on familiar and unfamiliar snacks

#### Liking

The PACE labels did not significantly change liking compared with non-PACE labels, for both familiar (26·1 (sem 0·5) *v.* 26·8 (sem 0·5)) and unfamiliar (21·1 (sem 0·8) *v.* 20·5 (sem 0·8)) snacks (*P*>0·05).

#### Consumption and prospective consumption

**Fig. 2:**
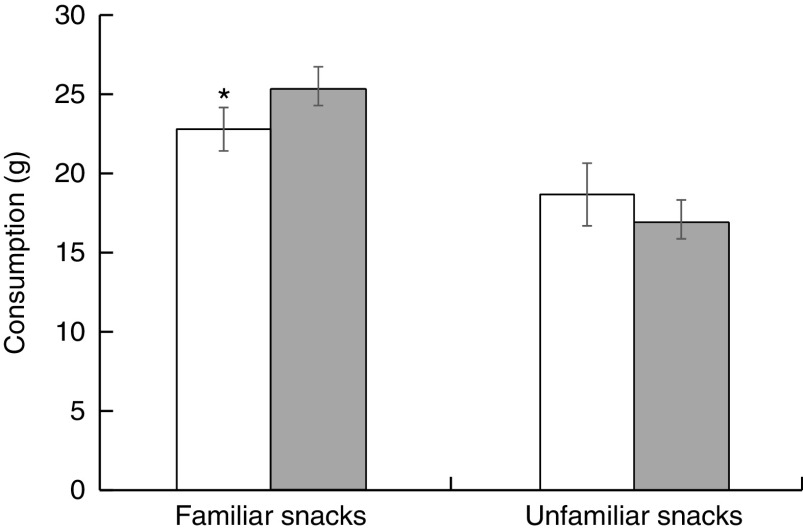
Mean consumption (in grams; with standard error of the mean represented by vertical bars) of PACE-labelled (PACE and PACE×2; 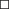) familiar (Honey Cashews and Nacho Cheese Shapes) and unfamiliar (Shrimp Peanut Crackers and Cheese Rice Crackers) snacks compared with non-PACE labelled (Blank and Fake; 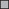) familiar and unfamiliar snacks among 153 university students (126 females, twenty-seven males, mean age 24·3 (sd 4·9) years) enrolled in an undergraduate nutrition degree at Deakin University, Australia. The PACE label decreased the consumption of familiar snack foods: *P<0·05 (PACE, physical activity calorie equivalent; PACE×2, PACE label with walking minutes doubled)

**Fig. 3:**
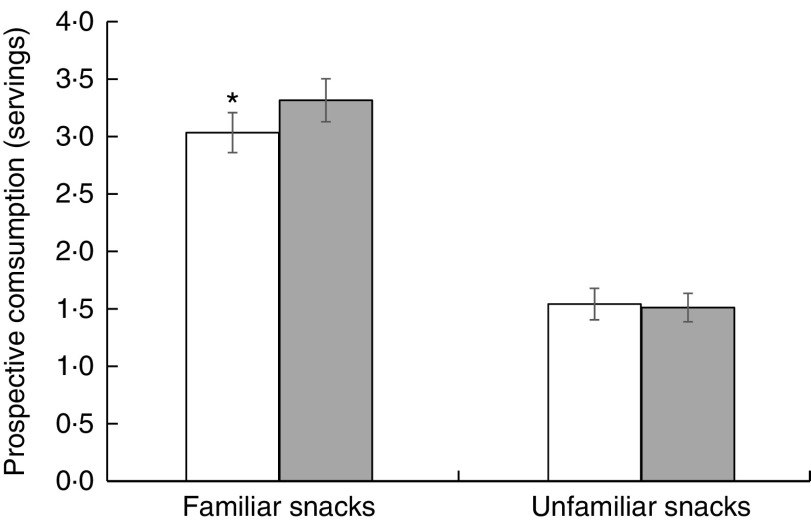
Mean prospective consumption (number of servings; with standard error of the mean represented by vertical bars) of PACE-labelled (PACE and PACE×2; 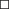) familiar (Honey Cashews and Nacho Cheese Shapes) and unfamiliar (Shrimp Peanut Crackers and Cheese Rice Crackers) snacks compared with non-PACE labelled (Blank and Fake; 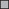) familiar and unfamiliar snacks among 153 university students (126 females, twenty-seven males, mean age 24·3 (sd 4·9) years) enrolled in an undergraduate nutrition degree at Deakin University, Australia. The PACE label decreased the prospective consumption of familiar snack foods: *P<0·05 (PACE, physical activity calorie equivalent; PACE×2, PACE label with walking minutes doubled)

As shown in Figs 2 and 3, the PACE labels decreased consumption of familiar snack foods by 9·9 % (22·8 (sem 1·4) *v.* 25·3 (sem 1·5) g, *P*=0·025) and prospective consumption by 9·1 % (3·0 (sem 0·2) *v.* 3·3 (sem 0·2) servings, *P*=0·027). Such pattern was not seen in unfamiliar snacks, with no significant difference in consumption (18·7 (sem 2·0) *v.* 16·9 (sem 1·5) g, *P*=0·684) or prospective consumption (1·5 (sem 0·1) *v.* 1·5 (sem 0·1) servings, *P*=0·799) between the PACE labels and non-PACE labels.

## Discussion

The purpose of the present study was to determine whether presenting the PACE label on discretionary snack foods influenced participants’ liking, prospective consumption and actual consumption of both familiar and unfamiliar snack foods compared with non-PACE labels.

It was found that the presence of the PACE labels on familiar snack foods resulted in participants sampling 9·9 % less (in grams) compared with the same snacks presented with non-PACE labels. Likewise, a 9·1 % reduction in prospective consumption (in servings) of familiar products was seen in the presence of the PACE label. Such pattern was not seen for unfamiliar products. No significant difference was found in the presence of the PACE label for liking, for both familiar and unfamiliar products.

In the present study, the amount of familiar products participants sampled was significantly influenced by the PACE label. This is unlikely to have been caused by liking, as no significant difference in liking was found with the presence of the PACE label. Potentially the PACE label acted as a cue to remind participants of the high energy content of the familiar snack foods, driving participants to sample less rather than affecting the liking. Some previous research has found FOP labelling to have an influence on taste perception and liking of foods^(^ ^35^ ^,^ ^36^ ^)^, with other studies, including the present study, finding no such effect^(^ ^37^ ^)^. Liking may have not been influenced by the PACE label as the snacks tasted within each snack type were in fact identical products; earlier studies have had similar findings^(^ ^37^ ^)^. Furthermore, as the PACE label only shows exercise information and no additional information pertaining to ingredients of the product, this may additionally explain why no significant difference was observed for liking. Although no change in liking was found in the presence of the PACE label, the reduction in consumption observed may have positive public health implications.

Currently 35 % of Australians’ daily energy intake consists of discretionary foods, with an estimated 10·5 % of this coming from discretionary snack foods such as chips/crisps, other salty snacks, cakes, sweet biscuits, ice cream and chocolate^(^ ^8^ ^)^. Positive energy imbalance of 209 kJ daily is calculated to increase the weight of an individual by approximately 2·2 kg per year^(^ ^38^ ^)^. To prevent this weight gain, an individual would need to eat, for example, only 10 g less of a discretionary salty snack each day to reduce energy intake by 200 kJ^(^ ^38^ ^,^ ^39^ ^)^. The results of the present study are promising and could potentially contribute to this small decrease in the consumption of certain discretionary snack foods. However, it needs to be noted that long-term studies are needed using a more representative population. Furthermore, the current study did not investigate whether the PACE label would impact physical activity levels of the participants. Previous research has found that 64 % of participants thought the PACE label would be ‘somewhat likely’ or ‘very likely’ to encourage them to partake in physical activity^(^ ^24^ ^)^. The effect the PACE label has on physical activity levels of participants is worth investigating in the future.

In addition to consumption and liking, measuring prospective consumption is also important, as it may give further insight into actual eating behaviour. A recent study found that prospective consumption of snack foods was related to actual consumption in an *ad libitum* consumption task^(^ ^40^ ^)^. Although the present study did not assess *ad libitum* consumption, it has been suggested that measuring consumption during laboratory taste tests is also a valid measure to investigate factors that may influence consumption^(^ ^41^ ^)^, such as FOP labelling. In the present study, a relationship was found between participants’ prospective consumption and actual consumption. It was found that the presence of the PACE label on familiar snack foods resulted in a 9·1 % reduction in prospective consumption and a 9·9 % reduction in how much participants sampled compared with the same snacks presented with non-PACE labels. The present study is the first to investigate the influence the PACE label has on participants’ prospective consumption and actual consumption of both familiar and unfamiliar discretionary snack foods. Furthermore, to our knowledge, past research has only investigated the influence of the PACE label on familiar, readily available fast-food menus^(^ ^24^ ^,^ ^27^ ^,^ ^31^ ^)^, snacks^(^ ^26^ ^,^ ^28^ ^)^ and sugar-sweetened beverages^(^ ^29^ ^,^ ^30^ ^)^. The influence the PACE label has on unfamiliar products has yet to be explored. Hypothetically, the influence of the PACE label on consumer behaviour may be different for familiar and unfamiliar products. This may be because nutritional information of products that consumers frequently purchase may be neglected^(^ ^28^ ^)^. Contrary to this, the present study found the PACE label to be effective at reducing consumption of familiar products. This finding is supported by previous studies that have found the PACE label to reduce selection and/or consumption of familiar foods^(^ ^24^ ^,^ ^26^ ^,^ ^27^ ^,^ ^28^ ^,^ ^31^ ^)^. However, the present study suggests that the PACE label does not seem to impact consumption of unfamiliar products. Previous research has found participants do pay attention to FOP labels on novel brands, which is important as attention is initially required for participants to then comprehend FOP labels^(^ ^42^ ^)^. However, it is possible that participants did observe the PACE label, but for the PACE label to then impact participants’ consumption, a sufficient amount of the snack foods needs to be consumed. Recall that in the present study participants consumed significantly less of the unfamiliar than of the familiar snack foods, or did not taste the unfamiliar snack foods at all. Therefore, it may not be that the results seen are due to the PACE label not having an impact on unfamiliar snacks, but that consumption of unfamiliar snacks was too low to detect a significant difference between the PACE and non-PACE labels.

### Limitations

The use of a university sample consisting of predominantly female, nutrition and food science students, with healthy BMI who are likely to have a high nutrition literacy and health awareness, reduces the external validity of the findings. However, previous studies have found that presenting a PACE-type label (presented as either minutes or miles to walk) to be effective in lowering the number of kilojoules ordered^(^ ^27^ ^)^ and consumed^(^ ^30^ ^)^ in populations with low kilojoule and numeracy literacy^(^ ^27^ ^)^. This indicates that the PACE label may be effective in both health-conscious, nutrition-literate populations and populations with lower nutrition and numeracy capabilities. The present study was conducted in a laboratory, which is not representative of the real-world scenario as variables such as cost, time constraints and other marketing information are not present which may impact consumers’ behaviour. One could argue that the 45 min tasting session was rather long, which could result in ‘mindless clicking’ without participants consciously processing the information provided. However, first, the tasting session was broken up by regular breaks between each snack food (2 min) and between sessions (5 min); and second, mindless clicking in the case of the present study is not necessarily detrimental to the results. Discretionary snack consumption has been suggested to be habitual behaviour that occurs with little thought^(^ ^43^ ^)^ and is influenced by cues in the environment^(^ ^44^ ^)^. Subtly changing the food environment by providing nudges (such as FOP labels) may make certain behaviours more likely^(^ ^45^ ^)^. It is thought that a lot of our behaviour occurs with little cognitive processing, but is triggered by factors in the environment without our direct awareness^(^ ^45^ ^)^. Providing FOP labels such as the PACE label is an example of a subtle nudge that is salient to participants. Additionally, in real-world food choice or consumption environments consumers are not told to look at specific information on labels when they are making decisions. Therefore, we decided it was not necessary to include attention checks throughout the tasting sessions. Furthermore, participants were not required to complete FFQ and/or food behaviour questionnaires. Therefore, it was unable to be determined whether the PACE label would have different effects in consumers with varying eating behaviours, such as restrained eating. Additionally, as we focused solely on savoury snack foods it is unclear whether the same effect would be found in sweet snack foods. Nevertheless, the study measured consumers’ prospective consumption and actual amount sampled, providing some possible understanding into the influence of the label in the real-world setting.

### Implications

The present study was the first to assess the effects of the PACE label on both prospective consumption and actual amount sampled for both familiar and unfamiliar discretionary snack foods. Previous studies have focused only on purchasing/selection^(^ ^29^ ^,^ ^30^ ^)^, hypothetical menu ordering of regularly consumed products^(^ ^24^ ^,^ ^27^ ^,^ ^28^ ^)^, or consumption of fast foods and snack foods^(^ ^31^ ^)^. If the PACE label reduces the amount consumers sample (and potentially consume) of discretionary snack foods by 9·9 % in the real world, as it did in the current study, there could be a potential public health benefit. However, longitudinal studies and studies in which *ad libitum* consumption is measured are needed to give a fair estimation of potential public health benefits.

## Conclusion

The PACE label appears to be a promising FOP label that could be used to decrease familiar discretionary snack food consumption in young, health-minded participants. Future research should aim to replicate these findings in a real-world scenario, using a representative sample of the population.

## References

[1] Buchmueller TC & Johar M (2015) Obesity and health expenditures: evidence from Australia. Econ Hum Biol 17, 42–58. 25637887 10.1016/j.ehb.2015.01.001

[2] Head GA (2015) Cardiovascular and metabolic consequences of obesity. Front Physiol 6, 32. 25713539 10.3389/fphys.2015.00032 PMC4322831

[3] Australian Bureau of Statistics (2013) 4338.0 – Profiles of Health, Australia, 2011–13. Height and weight. http://www.abs.gov.au/ausstats/abs@.nsf/Lookup/4338.0main+features212011-13 (accessed August 2017).

[4] Australian Bureau of Statistics (2015) 4364.0.55.001 – National Health Survey: First Results, 2014–15. Overweight and obesity. http://www.abs.gov.au/ausstats/abs@.nsf/Lookup/by Subject/4364.0.55.001~2014-15~Main Features~Overweight and obesity~22 (accessed July 2017).

[5] Hayes AJ, Lung TWC, Bauman A et al. (2017) Modelling obesity trends in Australia: unravelling the past and predicting the future. Int J Obes (Lond) 41, 178–185. 27671035 10.1038/ijo.2016.165

[6] Morgen CS & Sorensen TIA (2014) Obesity: global trends in the prevalence of overweight and obesity. Nat Rev Endocrinol 10, 513–514. 25048038 10.1038/nrendo.2014.124

[7] Hill JO, Wyatt HR, Reed GW et al. (2003) Obesity and the environment: where do we go from here? Science 299, 853–855. 12574618 10.1126/science.1079857

[8] Australian Bureau of Statistics (2016) 4364.0.55.012 – Australian Health Survey: Consumption of Food Groups from the Australian Dietary Guidelines, 2011–12. Key findings. http://www.abs.gov.au/ausstats/abs@.nsf/Lookup/4364.0.55.012main+features12011-12 (accessed August 2017).

[9] Niven P, Scully M, Morley B et al. (2015) What factors are associated with frequent unhealthy snack-food consumption among Australian secondary-school students? Public Health Nutr 18, 2153–2160. 25439182 10.1017/S1368980014002675 PMC10271753

[10] Savige GS, Ball K, Worsley A et al. (2007) Food intake patterns among Australian adolescents. Asia Pac J Clin Nutr 16, 738–747. 18042537

[11] Australian Bureau of Statistics (2014) 4364.0.55.007 – Australian Health Survey: Nutrition First Results – Foods and Nutrients, 2011–12. Table 9. http://www.abs.gov.au/AUSSTATS/abs@.nsf/DetailsPage/4364.0.55.0072011-12?OpenDocument (accessed August 2017).

[12] Cleobury L & Tapper K (2014) Reasons for eating ‘unhealthy’ snacks in overweight and obese males and females. J Hum Nutr Diet 27, 333–341. 10.1111/jhn.12169 24134077

[13] Bellisle F (2014) Meals and snacking, diet quality and energy balance. Physiol Behav 134, 38–43. 24657181 10.1016/j.physbeh.2014.03.010

[14] Arno A & Thomas S (2016) The efficacy of nudge theory strategies in influencing adult dietary behaviour: a systematic review and meta-analysis. BMC Public Health 16, 676. 27475752 10.1186/s12889-016-3272-x PMC4967524

[15] Sinclair SE, Cooper M & Mansfield ED (2014) The influence of menu labeling on calories selected or consumed: a systematic review and meta-analysis. J Acad Nutr Diet 114, 1375–1388.e1315. 25037558 10.1016/j.jand.2014.05.014

[16] Cecchini M & Warin L (2016) Impact of food labelling systems on food choices and eating behaviours: a systematic review and meta-analysis of randomized studies. Obes Rev 17, 201–210. 26693944 10.1111/obr.12364

[17] Morley B, Scully M, Martin J et al. (2013) What types of nutrition menu labelling lead consumers to select less energy-dense fast food? An experimental study. Appetite 67, 8–15. 23523666 10.1016/j.appet.2013.03.003

[18] Ellison B, Lusk JL & Davis D (2013) Looking at the label and beyond: the effects of calorie labels, health consciousness, and demographics on caloric intake in restaurants. Int J Behav Nutr Phys Act 10, 21. 23394433 10.1186/1479-5868-10-21 PMC3598881

[19] Temple JL, Johnson KM, Archer K et al. (2011) Influence of simplified nutrition labeling and taxation on laboratory energy intake in adults. Appetite 57, 184–192. 21569807 10.1016/j.appet.2011.04.018

[20] Harnack LJ, French SA, Oakes JM et al. (2008) Effects of calorie labeling and value size pricing on fast food meal choices: results from an experimental trial. Int J Behav Nutr Phys Act 5, 63. 19061510 10.1186/1479-5868-5-63 PMC2621234

[21] Watson WL, Chapman K, King L et al. (2013) How well do Australian shoppers understand energy terms on food labels? Public Health Nutr 16, 409–417. 22464021 10.1017/S1368980012000900 PMC10271533

[22] Maubach N, Hoek J & Mather D (2014) Interpretive front-of-pack nutrition labels. comparing competing recommendations. Appetite 82, 67–77. 25038407 10.1016/j.appet.2014.07.006

[23] Cramer S (2016) Food should be labelled with the exercise needed to expend its calories. BMJ 353, i1856. 27052519 10.1136/bmj.i1856

[24] Antonelli R & Viera AJ (2015) Potential effect of physical activity calorie equivalent (PACE) labeling on adult fast food ordering and exercise. PLoS One 10, e0134289. 26222056 10.1371/journal.pone.0134289 PMC4519110

[25] Viera AJ & Antonelli R (2015) Potential effect of physical activity calorie equivalent labeling on parent fast food decisions. Pediatrics 135, e376–e382. 25624379 10.1542/peds.2014-2902 PMC4306803

[26] Montford WJ, Peloza J & Goldsmith RE (2017) No pain, no gain: how PACE information attenuates consumption. J Consum Mark 34, 525–540.

[27] Dowray S, Swartz JJ, Braxton D et al. (2013) Potential effect of physical activity based menu labels on the calorie content of selected fast food meals. Appetite 62, 173–181. 23220355 10.1016/j.appet.2012.11.013

[28] Masic U, Christiansen P & Boyland EJ (2017) The influence of calorie and physical activity labelling on snack and beverage choices. Appetite 112, 52–58. 28082195 10.1016/j.appet.2017.01.007

[29] Scourboutakos MJ, Mah CL, Murphy SA et al. (2017) Testing a beverage and fruit/vegetable education intervention in a university Dining Hall. J Nutr Educ Behav 49, 457–465.e1. 28363803 10.1016/j.jneb.2017.02.003

[30] Bleich SN, Herring BJ, Flagg DD et al. (2012) Reduction in purchases of sugar-sweetened beverages among low-income black adolescents after exposure to caloric information. Am J Public Health 102, 329–335. 22390447 10.2105/AJPH.2011.300350 PMC3483987

[31] James A, Adams-Huet B & Shah M (2015) Menu labels displaying the kilocalorie content or the exercise equivalent: effects on energy ordered and consumed in young adults. Am J Health Promot 29, 294–302. 24575727 10.4278/ajhp.130522-QUAN-267

[32] Swartz JJ, Dowray S, Braxton D et al. (2013) Simplifying healthful choices: a qualitative study of a physical activity based nutrition label format. Nutr J 12, 72. 23742678 10.1186/1475-2891-12-72 PMC3681646

[33] Becker MW, Bello NM, Sundar RP et al. (2015) Front of pack labels enhance attention to nutrition information in novel and commercial brands. Food Policy 56, 76–86. 26417151 10.1016/j.foodpol.2015.08.001 PMC4582437

[34] Ainsworth BE, Haskell WL, Whitt MC et al. (2000) Compendium of physical activities: an update of activity codes and MET intensities. Med Sci Sports Exerc 32, 9 Suppl., S498–S504. 10993420 10.1097/00005768-200009001-00009

[35] Schouteten JJ, de Steur H, de Pelsmaeker S et al. (2015) Impact of health labels on flavor perception and emotional profiling: a consumer study on cheese. Nutrients 7, 10251–10268. 26690211 10.3390/nu7125533 PMC4690085

[36] Liem DG, Miremadi F, Zandstra EH et al. (2012) Health labelling can influence taste perception and use of table salt for reduced-sodium products. Public Health Nutr 15, 2340–2347. 22397811 10.1017/S136898001200064X PMC10271340

[37] Zandstra EH, Carvalho ÁHP & van Herpen E (2017) Effects of front-of-pack social norm messages on food choice and liking. Food Qual Prefer 58, 85–93.

[38] Kumanyika SK, Obarzanek E, Stettler N et al. (2008) Population-based prevention of obesity: the need for comprehensive promotion of healthful eating, physical activity, and energy balance: a scientific statement from American Heart Association Council on Epidemiology and Prevention, Interdisciplinary Committee for Prevention (formerly the expert panel on population and prevention science). Circulation 118, 428–464. 18591433 10.1161/CIRCULATIONAHA.108.189702

[39] Australian Government, Department of Health (2017) Discretionary Food and Drink Choices. https://www.eatforhealth.gov.au/food-essentials/discretionary-food-and-drink-choices (accessed November 2017).

[40] van den Akker K, Bongers P, Hanssen I et al. (2017) Validation of prospective portion size and latency to eat as measures of reactivity to snack foods. Appetite 116, 480–486. 28572066 10.1016/j.appet.2017.05.049

[41] Robinson E, Haynes A, Hardman CA et al. (2017) The bogus taste test: validity as a measure of laboratory food intake. Appetite 116, 223–231. 28476629 10.1016/j.appet.2017.05.002 PMC5504774

[42] Becker MW, Bello NM, Sundar RP et al. (2015) Front of pack labels enhance attention to nutrition information in novel and commercial brands. Food Policy 56, 76–86. 26417151 10.1016/j.foodpol.2015.08.001 PMC4582437

[43] de Vet E, Stok FM, de Wit JBF et al. (2015) The habitual nature of unhealthy snacking: how powerful are habits in adolescence? Appetite 95, 182–187. 26169248 10.1016/j.appet.2015.07.010

[44] Wilson AL, Buckley E, Buckley JD et al. (2016) Nudging healthier food and beverage choices through salience and priming. Evidence from a systematic review. Food Qual Prefer 51, 47–64.

[45] Kelly MP & Barker M (2016) Why is changing health-related behaviour so difficult? Public Health 136, 109–116. 27184821 10.1016/j.puhe.2016.03.030 PMC4931896

